# Safe surgical technique for associated acetabular fractures

**DOI:** 10.1186/1754-9493-7-7

**Published:** 2013-02-15

**Authors:** Takashi Suzuki, Wade R Smith, Cyril Mauffrey, Steven J Morgan

**Affiliations:** 1 Department of Orthopaedic Surgery, Kitasato University, School of Medicine, 1-15-1 Kitasato, Sagamihara, 252-0375, Japan; 2 MOTUS Mountain Orthopaedic Trauma Surgeons, Swedish Medical Center, 701 East Hampden Ave Suite 515 Englewood, Colorado, 80113, USA; 3 Department of Orthopaedic Surgery, Denver Health Medical Center, University of Colorado, School of Medicine, 777 Bannock Street Denver, Colorado, 80204, USA

**Keywords:** Acetabular fractures, Safe surgical technique, Acetabular fixation, Patient safety

## Abstract

Associated acetabular fractures are challenging injuries to manage. The complex surgical approaches and the technical difficulty in achieving anatomical reduction imply that the learning curve to achieve high-quality care of patients with such challenging injuries is extremely steep. This first article in the *Journal’s* “Safe Surgical Technique” section presents the standard surgical care, in conjunction with intraoperative tips and tricks, for the safe management of all subgroups of associated acetabular fractures.

## Introduction

The anatomic reduction remains the rationale for the surgical reduction and fixation of associated acetabular fractures, and is not different from simple acetabular fracture patterns. However, the surgical approaches required, the ability to receive an anatomic reduction, and the application of rigid internal fixation techniques is more complex.

Associated fractures, according to the Judet and Letournel classification [1], are comprised of two or more fracture lines that pass through the acetabulum and have complex geometries. With the exception of associated posterior column posterior wall pattern, the remaining associated fractures involve both the anterior and posterior columns. Due to the extensive involvement of both columns, the use of an extensile surgical exposure to visualize and reduce these fractures has been previously recommended. Compared to either the anterior or posterior approach, extensile exposures are associated with increased morbidity with respect to operative time, blood loss, infection, nerve injury, muscle weakness and heterotopic ossification. To minimize these complications a single non-extensile surgical exposure utilizing indirect reduction techniques have evolved and are utilized for the treatment of certain associated acetabular fractures [2].

The indications for surgical treatment are similar to the decision making for simple fractures. Significant displacement of fractures extending to the weight bearing dome of the acetabulum, incongruity of the hip joint, and hip instability generally require operative management [3]. To determine the involvement of the weight-bearing dome, the technique for roof arc measurement developed by Matta et al. [4,5] is helpful, in addition the assessment of the fracture relationship to the superior 10 mm of the acetabulum on axial CT scans corresponds to the roof arc measurement technique of Matta [4]. More recently the use of three-dimensional CT may provide a more accurate assessment of the involvement of the weight bearing subchondral arc of the acetabulum.

It is becoming clear that fractures of the acetabulum despite anatomic or near-anatomic reduction can potentially lead to altered stress distribution with the potential for the development of post traumatic arthritis. In some cases, displacement of less than 2 mm can be considered for nonoperative treatment and a reasonable outcome anticipated [3]. However, this criteria alone should not determine the surgical decision to operate without consideration of other confounding factors that may influence the clinical result including but not limited to: the existence of loose bodies, gaps, fractures of the femoral head, and local soft tissue conditions. In certain situations such as advanced age, patients other choices may be considered such as primary arthroplasty or secondary arthroplasty following percutaneous screw fixation or limited exposure internal fixation [6].

These are difficult fractures and the surgeon’s experience level should also be taken into consideration when considering operative fixation, as experience often increases the likelihood of the surgeon obtaining an anatomic or near anatomic reduction. Regardless of surgeon experience, one must have a good understanding of the three dimensional anatomy of the pelvis and acetabulum, the fracture configuration and be comfortable with the techniques, and equipment required to treat these injuries. It must be recognized that the prognosis is poor for patients who receive an inadequate surgical reduction when compared to those who are treated conservatively with similar fracture displacement [7].

Surgery for these fracture patterns should be performed under ideal circumstances with an experienced supporting ancillary staff of nurses, anesthetists, and scrubbed assistants. In general acute surgery within the first 48 hours of injury should be avoided in most cases to prevent excessive bleeding associated with the acuteness of the injury. In general operative fixation in the first three weeks following injury is satisfactory and does not lead to an escalation in surgical care or expansion of the surgical approaches required to achieve reduction [7]. It is clear, however, that the fracture reduction and associated co-morbidity with delayed surgery is avoided when surgical reduction and fixation is performed in the first 5 to seven days following injury. Surgical delay beyond three weeks is associated with a diminished prognosis secondary to organization of the fracture hematoma, soft tissue contracture and callus formation [7].

The perioperative planning and set-up may take into account a number of variables that depend on surgeon experience and preference. The universal use of a traction table is still controversial. Certainly the intraoperative traction of affected lower extremity is essential, but traction table devices may limit full motion of the extremity and prevent visualization in some positions either directly or with fluoroscopy. Alternatives to the use of the fracture table include intraoperative placement of a Schanz pin into the proximal femur for manual distraction of the joint. Intraoperative fluoroscopy is usually recommended to confirm the adequacy of the reduction, and extra-articular placement of the fixation.

The lateral view is the most effective view to confirm that the hardware is extra-articular. More importantly with the x-ray beam oriented in a linear array with the screw, extra-articular placement can always be confirmed or denied on this alone. Additionally, ranging the hip joint intraoperatively and checking the range of motion would help to find intra-articular screw misplacement, remaining instability, and malreduction of the fragments.

### Associated posterior column and posterior wall fractures

#### Take home message for safe surgical technique

•*Prone or lateral positioning; the patients’ hip should be extended and the knee flexed to reduce tension on the sciatic nerve*

•*Prone positioning will allow mechanical traction and facilitate reduction through gravity. External rotation of the hip will be possible, facilitation the reduction of a displaced posterior wall fragment.*

•*Retractors placed in the sciatic notches should be released as often as possible to prevent lengthy compression against the sciatic nerve*

•*Posterior wall capsular attachments should be maintained to prevent devascularization*

•*The superior gluteal neurovascular structures can be injured from excessive retraction of the abductor muscle mass. It is recommended to not use a very long plate and to keep the hip abducted (2 screws proximal and distal to the hip joint are usually sufficient)*

•*Always confirm that no screws are penetrating in the hip joint by using Judet views, if in doubt, reposition the screws in a different angle*

#### Surgical approach and patient positioning

This is the only associated fracture that does not involve both anterior and posterior columns and is best visualized utilizing the Kocher-Langenbeck approach. The patient can be positioned lateral or prone. Lateral positioning on a radiolucent table has the advantage of not requiring the use of a fracture table or the unfamiliarity of operating in the prone position. Reduction aids like a femoral distractor or manual traction can aid in the reduction of the fracture or visualization of the joint. The main disadvantage of this technique is the unreliable nature of the manually applied traction, the potential for undue tension on the sciatic nerve and the increased risk of sciatic nerve injury. Another major challenge to reduction is related to the persistent displacement of the posterior column as the result of gravity that can not be eliminated in this position. Prone positioning in traction offers the main advantage of gravity elimination and aids in the reduction of the posterior column [8]. The leg can be held flexed at the knee and extended at the hip to avoid traction on the sciatic nerve greatly reducing the chance of nerve injury. Controlled lateral traction can also be applied to help visualize the joint surface through the window of the posterior wall fracture after the posterior column has been reduced [9].

#### Operative procedure

**Figure 1:**
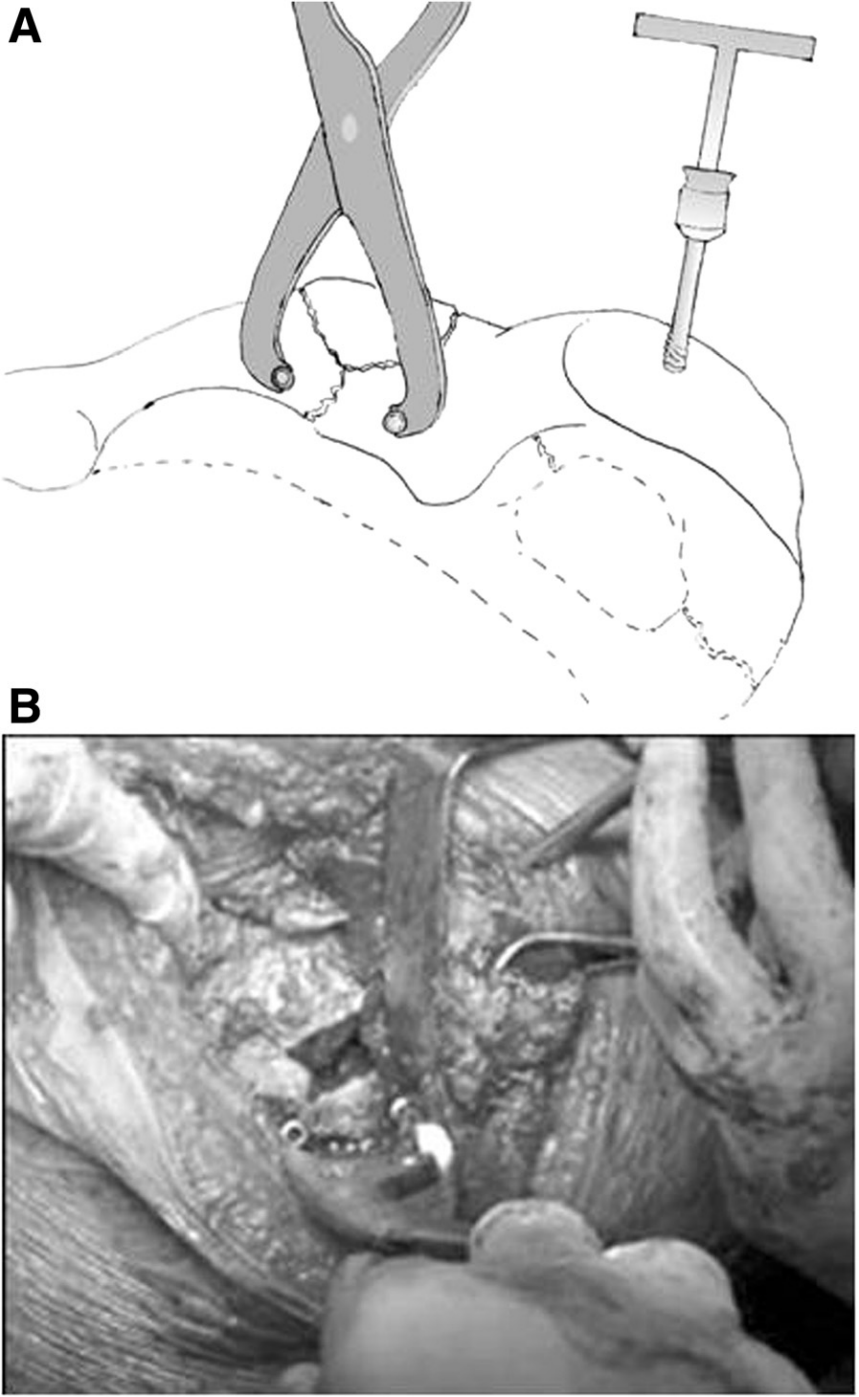
**Temporary two-screw technique to facilitate fracture reduction using a Faraboeuf or Jungbluth reduction clamp.** (**A**) Screws applied along with the great sciatic notch in order not to preclude plate placement. (**B**) Intraoperative view of screws and clamp.

Reduction of the posterior column fracture provides for a stable surface to reduce the posterior wall fracture. Thus, the posterior column fractures should be addressed first. Following the surgical approach and exposure of the retroacetabular surface and the lesser and greater sciatic notches including the ability to digitally palpate the quadrilateral surface, reduction of the posterior column can be undertaken. Care must be taken with retraction of the sciatic nerve. During this procedure retractors should be removed or retraction relaxed frequently to allow the nerve to have periods that are tension free. When work on the fracture surfaces is not being undertaken, the retractors should be released. Inexperienced assistants may not recognize this necessity, and it is incumbent on the operative surgeon to make sure this occurs. The fracture is generally displaced medially and rotated on the soft tissue attachment in the area of the ischium. Rotational control can be obtained by using a 5 or 6 mm Schanz screw inserted into the ischium. A universal T-handle can then be attached to the Schanz pin to serve as a handle to assist in derotating the column and to some extent reducing its medial displacement. The superior aspect of the fracture following derotation can be potentially reduced with several different types of clamps including the angled jaw clamp, a Weber clamp, or a Fareboeuf clamp placed directly perpendicular to the fracture line. Alternatively, a bone hook can be introduced through the notch to reduce the fracture, but it requires continuous traction pending placement of a lag screw or a clamp. If these reduction maneuvers fail, the reduction can be obtained using Faraboeuf or Jungbluth clamps applied by means of temporary screws, what is called the two-screw technique (Figure 1). The former is particularly helpful in reducing gap displacement with minimal rotational abnormality while the later is useful for both significant medialization, rotational and gap displacement. The problem with the clamps and their increasing size is the difficulty they present with the introduction of plate fixation or lag screw placement secondary to the occupation of the available operative space by these tools. The reduction is confirmed by digital palpation of the quadrilateral surface and the greater sciatic notch. The intra-articular surface may be directly visualized by reflecting the posterior wall fragments in continuity with the joint capsule and by distracting the hip joint. By internal rotation, the hip may be re-dislocated and washed off all small fragments of debris. After posterior column reduction, stabilization is achieved with a short 3.5 mm reconstruction plate positioned near the greater sciatic notch. A 3.5 mm lag screw from the posterior column through the fracture line alongside the deep aspect of the quadrilateral surface may facilitate the removal of clamps and maintenance of the reduction during the subsequent plate fixation.

**Figure 2:**
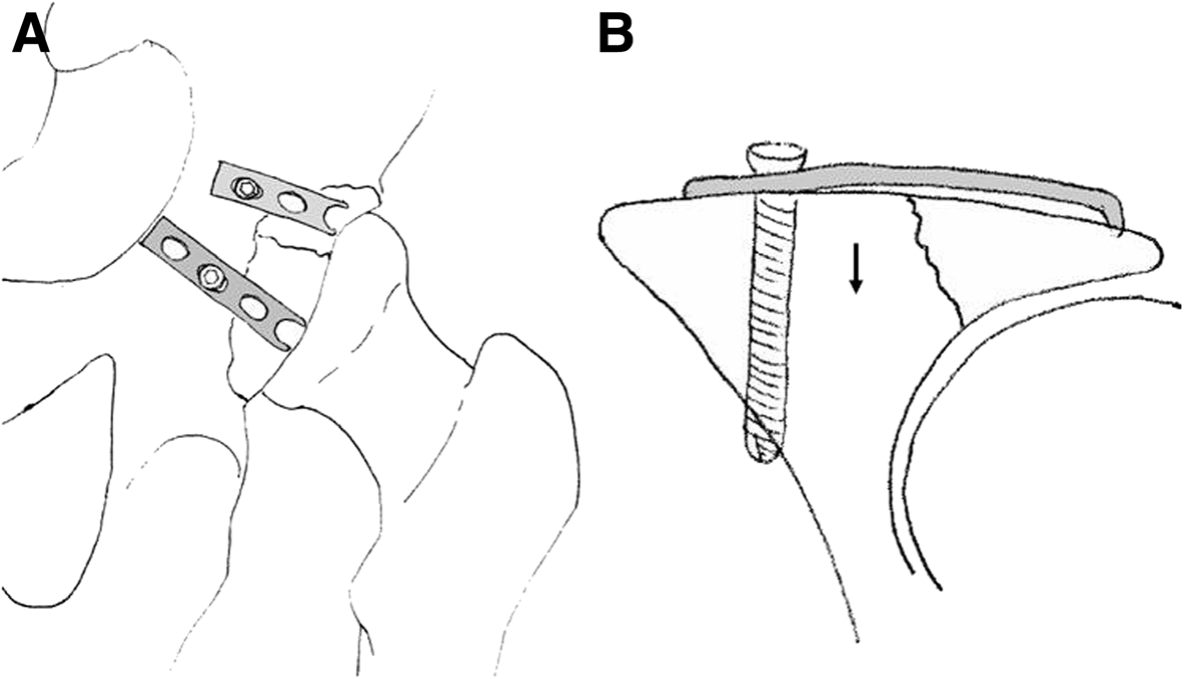
**Schematic drawing on the use of spring plates.** (**A**) One third tubular plate placed over the posterior wall fragments. (**B**) A spring plate pushes and holds fracture fragments which are deemd to small for fixation with a screw. The plate must be oriented so as not to interfere with the joint.

The second step is the reduction of the posterior wall. Throughout the procedure, care must be taken to preserve the capsular attachment to all posterior wall fragments to avoid excessive devascularization. A suture can be placed in the capsule to facilitate retraction and visualization of the posterior column reduction. The traction should be released and the femoral head is used as a template for the reduction of the posterior wall fracture. Marginal impaction, when present, requires elevation of the articular surface by a curved chisel or osteotome with additional support obtained by bone grafting the void left following elevation of the impacted segment. The autologous bone graft can be obtained from the greater trochanter. Free pieces of articular surface should then be relocated in appropriate position utilizing the femoral head as a guide. The main wall fragments can then be correctly reduced with the ball spike pusher, followed by provisional fixation with K-wires. Fixation should consist of buttress plating with the adjunctive lag screw fixation when fragment size is sufficient. Lag screws alone do not provide sufficient stabilization. A 3.5 mm reconstruction plate, or acetabluar specialty plates are the traditional implants of choice for buttress fixation. If the fragments are comminuted, small or very peripheral then a spring plate can be applied (Figure 2). This is achieved by cutting a one-third tubular plate through the end hole and placing it over the fragment. The spring plate is slightly over-contoured so that when the reconstruction plate is applied over the spring plate, the captured fragments are held firmly in position. Application of the buttress plate requires the distal portion of the plate to extend low enough on the ischium to permit the most distal screw to be placed into the ischiopubic ramus. Screw placement in the central area of the posterior column is avoided to prevent intra-articular placement. Generally, 2 distal screws and 2 proximal screws are sufficient for adequate buttress fixation. Visualization of the proximal part of the plate by muscle retraction may be obtained by careful placement of a Hohmann retractor hammered into the intact ilium. However, the superior gluteal neurovascular structures can be injured from excessive retraction of the abductor muscle mass. It is recommended to not use a very long plate and to keep the hip abducted. At the end of the operation, it is advisable to check for intra-articular screws by both moving the hip while listening for audible crepitance and by using fluoroscopy.

#### Tips and tricks

**Figure 3:**
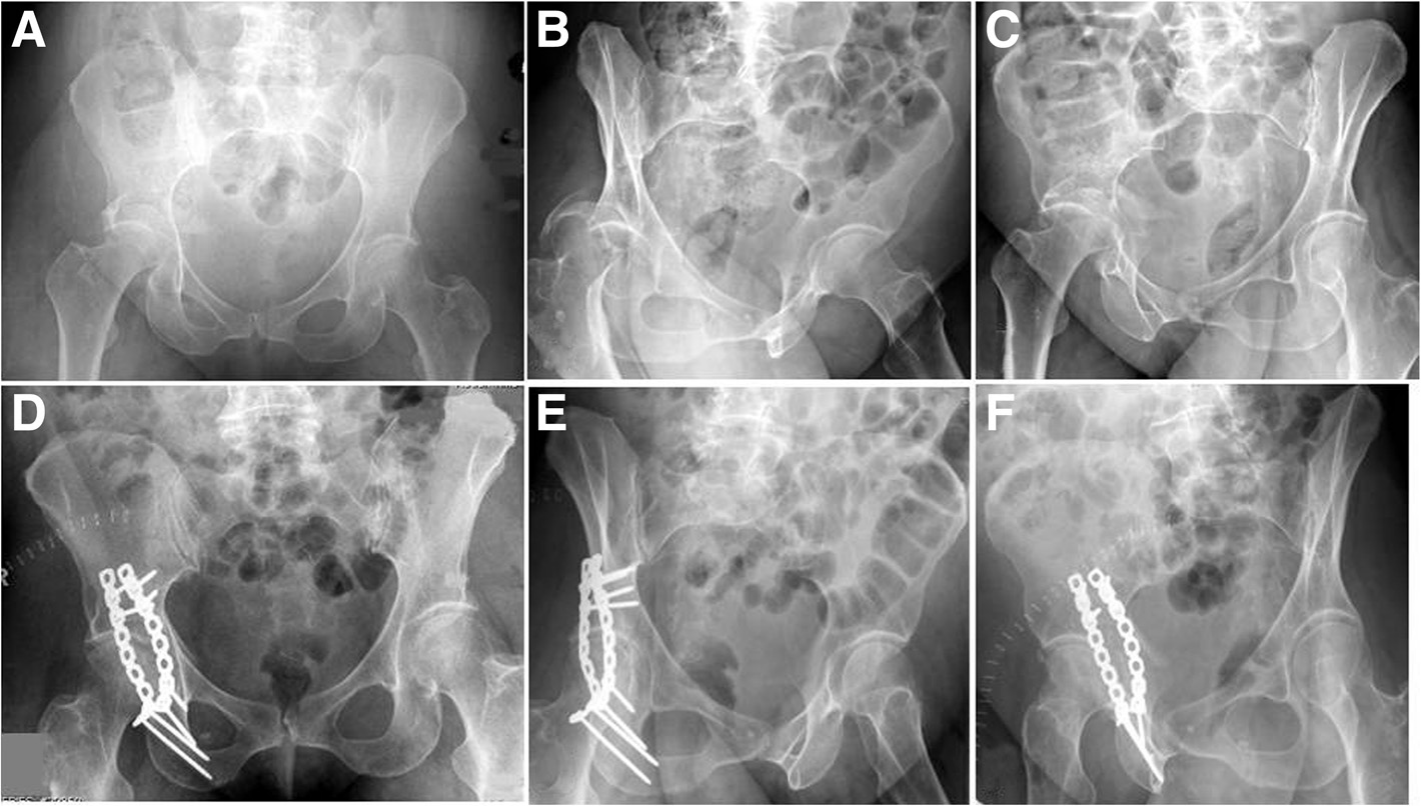
**Example of an associated posterior column and posterior wall fracture treated with two 3.5 mm reconstruction plates.** (**A-C**) Preoperative X-rays (a.p., obturator oblique, iliac oblique). (**D-F**) Postoperative X-rays (a.p., obturator oblique, iliac oblique).

The two posterior plates should be separated from each other as far as possible as the close placement of these two plates precludes not only the sufficient buttress effect on the posterior wall fragments but also the attainment of sufficient mechanical strength of the posterior column fixation (Figure 3). It is especially important to place the buttress plate accurately over the main portion of the posterior wall fragment and just outside the margin of the hip joint. A slight undercontouring of this plate will direct compressive forces across the fragment and can buttress the entire posterior wall firmly.

### Associated anterior column and posterior hemitransverse fractures

#### Take home message for safe surgical technique

•*Safe positioning of the patient in the supine position with option for traction of the affected extremity is recommended.*

•*The ilio-inguinal or modified Stoppa approaches are valid options. The latter approach when the anterior column fracture is low and does not involve the iliac wing.*

•*Structures at risk during the Stoppa approach include the obturator vessels and nerve (because of their direct contact to quadrilateral surface) and the iliolumbar vessels. A corona mortis is also present in 10-30% of cases and is at risk during both the Stoppa and the medial window of the Ilio-inguinal approach.*

•*Reduction technique should proceed in a centripetal direction, towards the articular surface.*

This fracture pattern is comprised of an anterior column fracture with an additional posterior half of a pure transverse fracture. This pattern may be considered as an atypical or transitional fracture from T-shaped to both column fractures. In general the posterior column portion of the fracture remains non or minimally displaced, and the displacement of the femoral head is associated with the position of the anterior column. The operative treatment is less difficult than that of a both column or T-Type fracture, and the surgical approaches are generally anterior.

#### Surgical approach

These fractures are best fixed utilizing an anterior based surgical approach. Of the anterior approaches, the ilioinguinal approach is usually used. If there is one large fragment comprising the anterior part of the iliac wing and the distal fracture line exists around the iliopectineal eminence, the iliofemoral approach can be utilized. The modified Stoppa approach can be utilized, when the anterior column fracture is low and does not involve the iliac wing. In general, however, this fracture pattern of the anterior column in this grouping is rare and the modified Stoppa rarely utilized [10]. The patient can be placed supine on a fracture table or supine on a radiolucent table. Skeletal traction via the distal femur and lateral displacement traction via the proximal femur utilizing a fracture table or manual traction will help aid the reduction process.

The Swiss group from Berne [11] has recently described a case series of 20 patients treated with a single para-rectal extra-peritoneal approach. This approach involves dissection of the external iliac vessels, the inferior epigastric vessels, and the spermatic cord or round ligament with five separate windows described allowing full exposure of the quadrilateral plate and an intra-articular view through the displaced fracture of the quadrilateral plate.

#### Operative procedure

The reconstruction of the anterior column begins with the reduction of the iliac fragments to portions of the intact pelvis, proceeding sequentially toward the articular surface. The anterior column is usually externally rotated and the reduction is initiated by derotating the anterior column with a ball spike pusher placed just above the pelvic brim on the distal to middle aspect of the inferior portion of the anterior column fragment. A Faraboeuf clamp can be placed at the iliac crest or between the anterior superior and inferior iliac spines to further assist in the derotation of the anterior column. The first point of reduction should occur at the iliac crest. A small window in a subperiosteal fashion is developed so digital palpation of the outer table of the iliac wing can be performed. A pointed reduction clamp can compress the iliac crest together at the fracture line. When significant purchase cannot be obtained, the grip of the reduction forceps can be improved by drilling two separate holes on either side of the fracture for the clamp tips. Alternatively, a Faraboeuf clamp placed on the iliac crest after two screws are placed parallel to the fracture line can be utilized to obtain the same goal. Once the iliac crest is stabilized, compression at the pelvic brim fracture line and final reduction can be obtained by placing a small angled jaw clamp across the fracture line typically via the second window of the ilioinguinal exposure. An alternative to clamp placement is final reduction with the ball spike pusher at the level of the pelvic brim and provisional fixation with divergent K-wires. Internal fixation is commenced at the iliac crest. The fracture line at this level can be stabilized by using one or two 3.5 mm lag screws placed between the tables of the iliac crest. If inner table screws are not possible a pelvic reconstruction plate can be contoured to the inner table of the crest, or the crest itself, and fixed with bicortical screws. Placement of the plates directly on the crest is generally avoided secondary to the associated hardware irritation that becomes prevalent with time especially on the anterior aspect of the iliac crest. Lag screw fixation may provide more stable fixation than a 3.5 mm reconstruction plate applied to the iliac crest alone [12]. Fixation should then proceed closer to the pelvic brim. Some fracture patterns lend themselves to screw fixation alone. An additional inner table screw can be placed from between the anterior superior and inferior iliac spines towards the sciatic buttress. Assuming the posterior hemitransverse component remains reduced, an additional two screws are then placed form the pelvic brim superior to the acetabulum directly in to the posterior column and when possible in to the ischium passing between the acetabulum and the greater and lesser sciatic notches. If the posterior column requires reduction, it can be reduced as described below prior to placement of the lag screws. In good quality bone with a high anterior column component this amount of fixation is likely sufficient and plate fixation can be avoided. If the posterior column requires further reduction, a single screw can often be placed from the anterior column at the level of the posterior aspect of the pelvic brim to the area of the sciatic buttress avoiding the anterior column. Alternatively, the anterior column can be buttressed with a long 3.5 mm reconstruction plate, which is usually 12 to 14 holes long. This is contoured along the pelvic brim, across the iliopectineal eminence to the pubic tubercle and the body of the pubis. Cortical screws are then placed in the area of the sciatic buttress aiding in the reduction of the anterior column. Additional screw fixation is avoided until the posterior column is reduced. The symphysis should not need to be routinely incorporated in to the plate construct. It is essential that the plate be perfectly contoured; otherwise, tightening down the plate may result in malreduction of the column fracture. It is essential that screws do not capture a malreduced posterior column, preventing further reduction.

**Figure 4:**
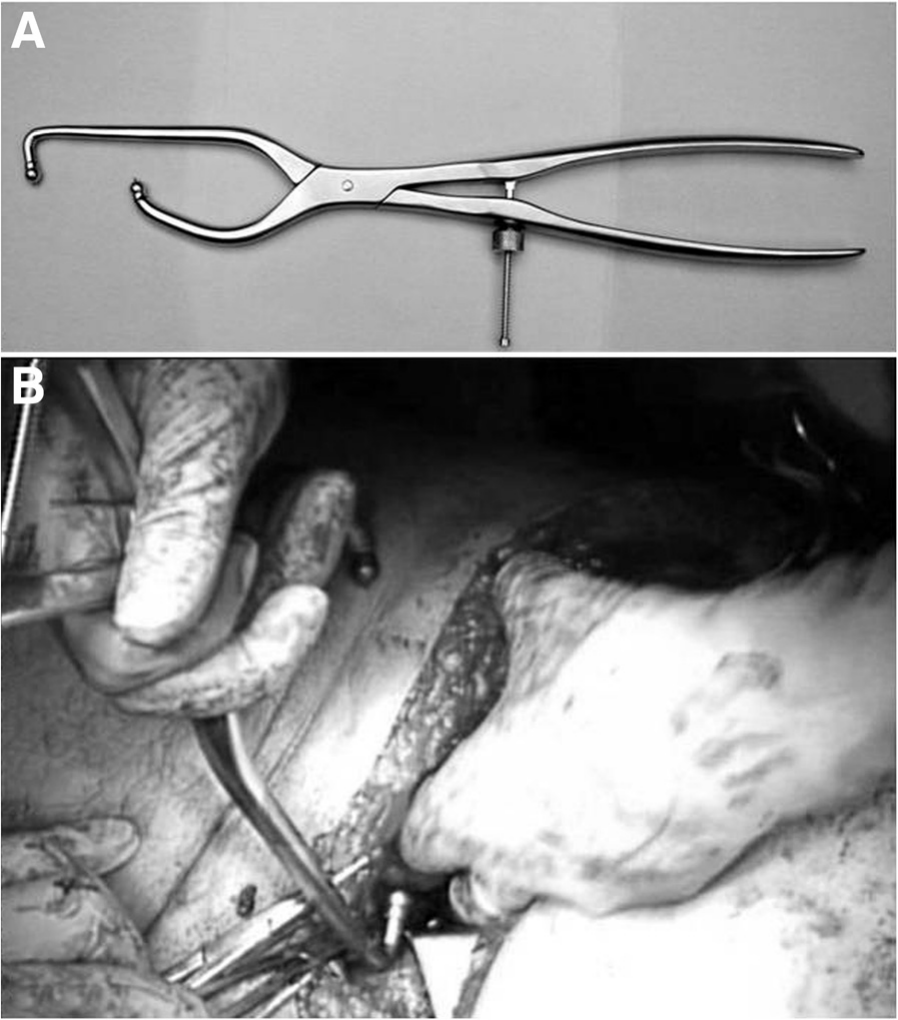
Asymmetric pelvic reduction clamp (A). Intraoperative view using the asymmetric clamp (B).

**Figure 5:**
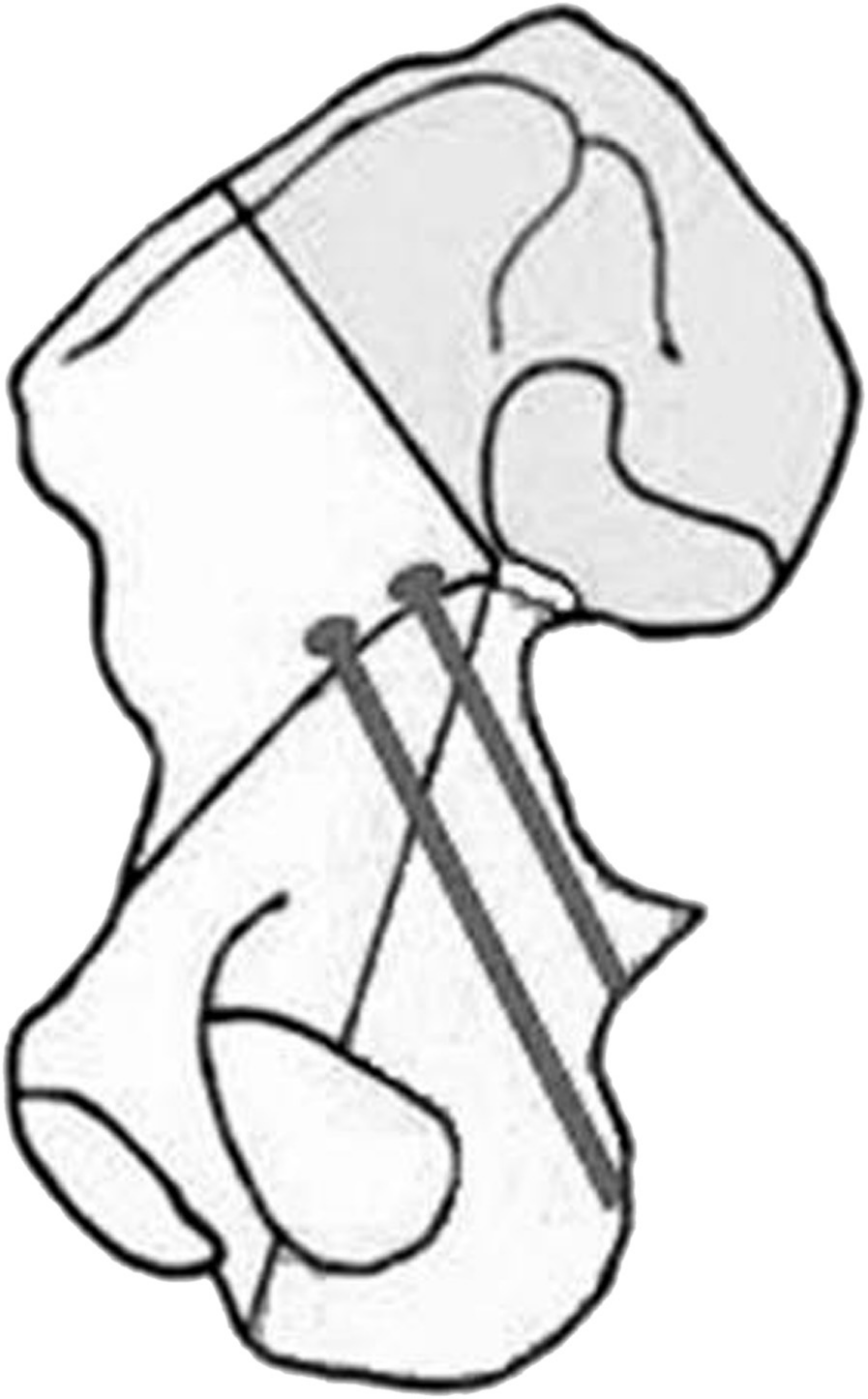
**Schematic model of lag screw positioning from the pelvic brim directed to the posterior column.** Small fragment (3.5 mm) cortical screws are usually used at a length of more than 80 mm.

The next step is the reduction of the posterior column. If the hemitransverse fracture line is located low, the posterior column is already reduced or slightly displaced and may be neglected after the reduction of the anterior column. If the fracture line is high, it is not automatically reduced. When using ilioinguinal approach, the reduction should be indirect through the first or second window. The displaced posterior column is usually rotated internally and the reduction may be possible with the jaws of an asymmetrical clamp applied, between the outer surface of the anterior inferior iliac spine and the other on the quadrilateral surface attached to the posterior column (Figure 4). A small bone hook or coaxial pelvic clamp, gently slid down the quadrilateral surface, can help with manipulation of the posterior column. The reduction is maintained by 3.5 mm screws which can be inserted from the posterior or middle third of the upper aspect of the pelvic brim, either apart from the plate or through the holes of the plate (Figure 5). These screws start at the pelvic brim superior to the acetabulum and are directed from proximal to distal into the posterior column paralleling the quadrilateral surface, aiming for the ischial spine. The screw length is usually more than 80 mm and often up to 110 mm. Care must be taken to avoid intra-articular placement of these screws; therefore, it is important to appreciate the location of the acetabulum relative to the fixed pelvic landmarks, that is, inferior to the anterior inferior iliac spine and under the iliopubic eminence. Additional fixation of the plate to the pubic symphysis can now be undertaken completing the case.

#### Tips and tricks

**Figure 6:**
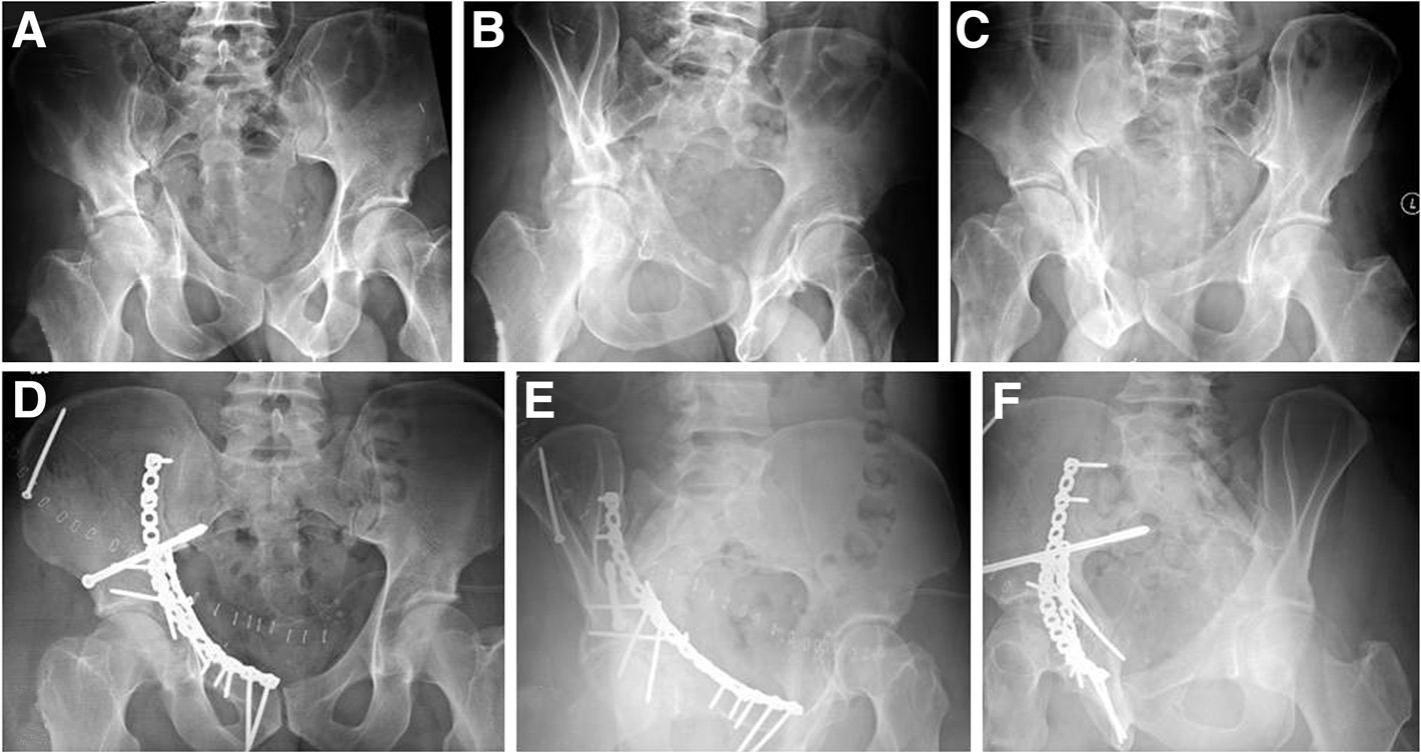
**Example of an anterior column and posterior hemitransverse fracture treated through a modified ilioinguinal approach, using 3.5 mm reconstruction plates and a 7.3 mm cannulated screw.** (**A-C**) Preoperative X-rays (a.p., obturator oblique, iliac oblique). (**D-F**) Postoperative X-rays (a.p., obturator oblique, iliac oblique).

**Figure 7:**
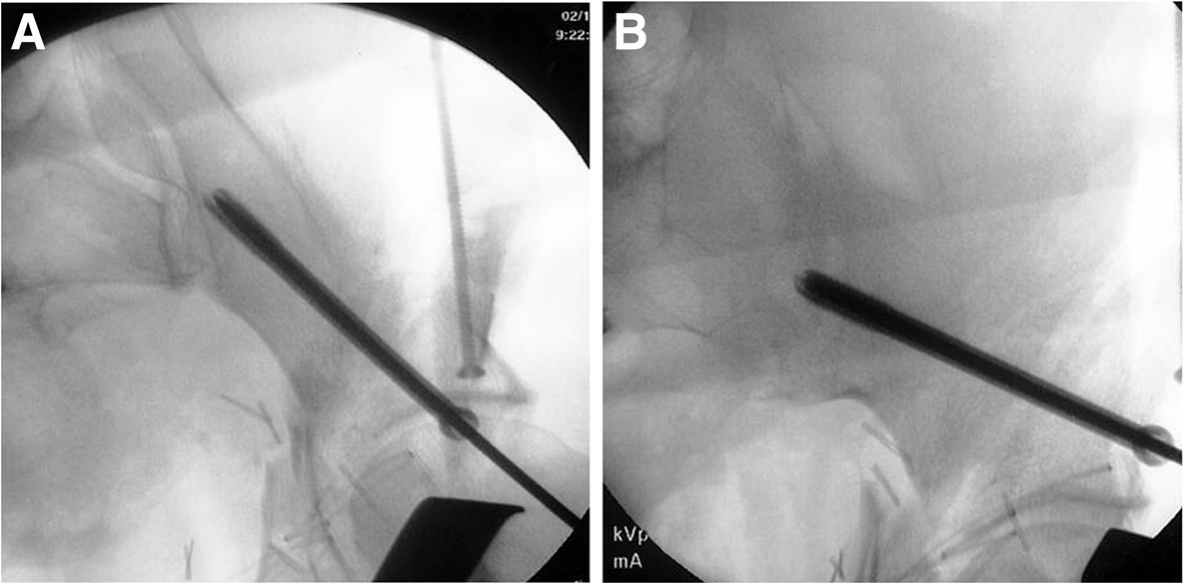
**Intraoperative fluoroscopic images of cannulated screw placement from the anterior inferior iliac spine toward the superior posterior iliac spine.** (**A**) Inlet-obturator oblique view. (**B**) Iliac oblique view.

After the reduction of the anterior column, instead of K-wires, 6.5 to 7.5 mm cannulated screw placement from the anterior inferior iliac spine though the iliac fracture site toward the superior posterior iliac spine can provide sufficient stability during the reduction of the posterior column (Figure 6). This screw fixation was first reported for iliac wing fractures of the pelvic ring, but is also useful for the fixation of the acetabular fractures that involve the anterior column. Its position in the ilium is checked using intraoperative fluoroscopy on both the inlet-obturator oblique view and on the iliac oblique view [13] (Figure 7).

### Associated transverse and posterior wall fractures

#### Take home message for safe surgical technique

•*The safety and choice of approaches is determined by the location of the transverse fracture: infra-tectal fractures can be dealt with via a KL approach while juxta and trans-tectal will require anatomical reduction usually via an anterior approach.*

•*Lateral decubitus will allow for a two incision technique*

•*The sciatic nerve is at risk, especially when attempting an indirect reduction of the anterior fracture line through a KL approach*

•*When fixation of the transverse component is done through a posterior to anterior screw (anterior column screw), over penetration of the anterior column (anteriorly) with the drill or screw can damage the femoral neuro-vascular bundle or the external iliac vein or artery if the over penetration is through the superior cortex of the anterior column. Iliac and obturator oblique (Judet) views are crucial during this process.*

The association of a transverse fracture with a posterior wall fracture is not uncommon. The position of the transverse component of the fracture in relationship to the weight-bearing dome of the acetabulum will dictate the surgical approach and the subsequent positioning of the patient.

#### Surgical approach

The presence of a posterior wall fracture will always require the use of a posterior approach but this alone does not necessarily preclude the use of the anterior approach. Infratectal transverse fractures can be treated with a posterior Kocher-Langenbeck approach alone. Transtectal and juxta tectal fractures require anatomic reductions for optimal outcomes [14,15]. While many of these can be treated utilizing the posterior Kocher-Langenbeck approach, some may benefit from the use of an extensile incision or two incision technique to insure anatomic reduction of the anterior portion of the transverse component of the fracture. This can be facilitated by utilizing a two incision approach to acetabular reduction and fixation, or the use of the extended illofemoral approach [16]. Secondary to the morbidity of the extended illiofemoral approach the authors prefer a simultaneous two incision approach in the lateral position.

#### Operative procedure

These fractures are best treated by approaching the reduction of the transverse fracture first, utilizing the posterior wall fracture as a window to the joint to directly visualize the quality of the reduction, before fixation of the posterior wall fracture.

**Figure 8:**
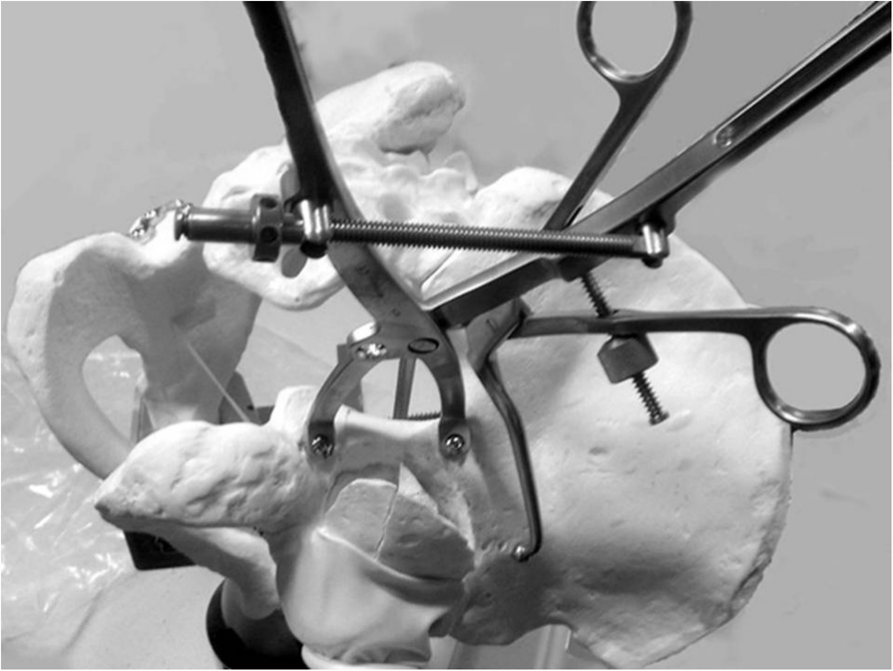
**Pelvic saw bone model of an associated transverse and posterior wall fracture.** The reduction is temporarily maintained by using a two-screw technique with a Jungbluth clamp (see Figure 1) and an angled pelvic reduction clamp through the greater sciatic notch, placed on the quadrilateral surface.

The reduction is carried out in a fashion similar to that in a posterior column fracture. The inferior fragment is manipulated by the appropriate pelvic clamps while rotation is controlled by a Schanz screw inserted into the ischial tuberosity. Traction of the affected lower extremity can help this manipulation. The reduction is temporarily maintained by a Faraboeuf or Jungbluth clamp using a two-screw technique (Figure 8). The anterior reduction is confirmed by digital palpation of the quadrilateral surface to the iliopectineal line. If the anterior column is still displaced, then it is likely due to rotation of the fragment and not from simple inward displacement. This is corrected with a Schanz screw or an angled pelvic reduction clamp, with one jaw on the proximal intact ilium and the other jaw through the greater sciatic notch placed on the quadrilateral surface just below the pelvic brim of the anterior column (Figure 8). During this process, the sciatic nerve should be monitored and undue tension avoided. If reduction of the anterior column portion of the acetabulum is felt to be less than satisfactory, an anterior approach utilizing either the illioinguinal or illiofemoral exposure can be employed. The anterior portion of the fracture can be directly visualized and generally reduced utilizing a ball spike pusher, Faraboeuf clamp, or antiglide plate.

Once reduction is obtained the inferior segment of the transverse component can frequently be provisionally fixed with a single lag screw. The screw placed either from the intact ilium just above the angle of the greater sciatic notch to the distal posterior column or from the angle of the greater sciatic notch to the intact ilium may be effective. A single posterior plate can also secure this portion of the transverse fracture pattern. The plate should be placed along the margin of the greater sciatic notch where the plate does not preclude the reduction and fixation of the posterior wall fracture. This plate should be overcontoured to achieve compression of the anterior column segment. A long lag screw placed down to the anterior column can be placed from the superior aspect of the retroacetabular surface into the anterior portion of the fracture. The starting point for this screw is approximately 3 to 4 cm above the acetabulum along with the anterior pillar of the iliac wing. This posterior-to-anterior lag screw is inserted across the obliquity of the transverse fracture line into the anterior column. This screw runs parallel to the quadrilateral surface, taking purchase in the anterior column. Its position in the anterior column is checked using the obturator oblique and iliac oblique views intraoperatively. It is important to avoid excessive anterior penetration with the drill bit to prevent damage to the femoral vessels. If a two incision approach is utilized, placement of the posterior to anterior screw can frequently be directly visualized. Alternatively, an anterior plate can also be utilized in these situations to reduce and secure the anterior column portion of the fracture with a plate.

**Figure 9:**
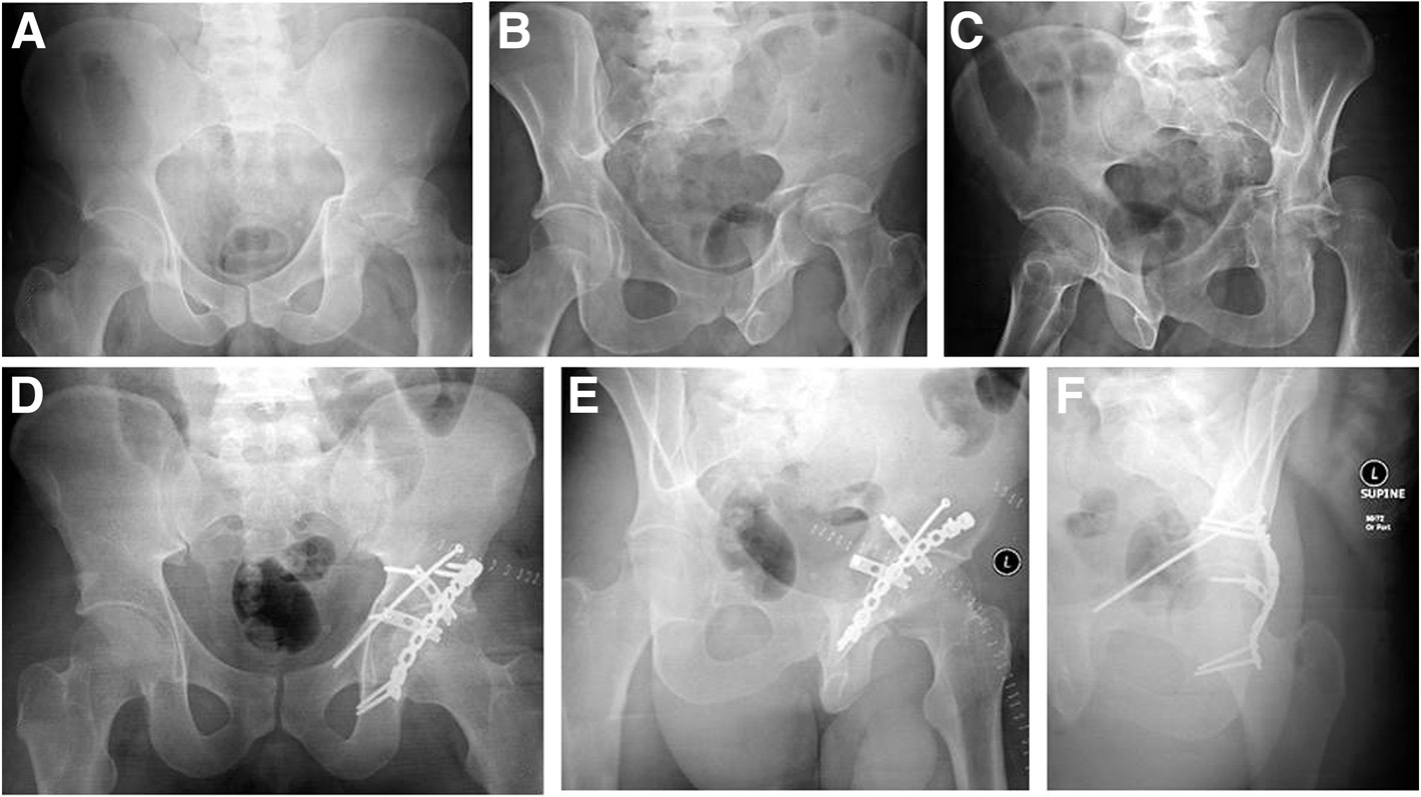
**Example of an associated transverse and posterior wall fracture treated through Kocher-Langenbeck approach, using a 3.5 mm reconstruction plate, two third-tubular spring plates, and a 3.5 mm anterior column screw.** (**A-C**) Preoperative X-rays (a.p., obturator oblique, iliac oblique). (**D-F**) Postoperative X-rays (a.p., obturator oblique, iliac oblique).

The next step is the reduction of the posterior wall. The principle is the same as that in the associated posterior column and posterior wall fractures. Traction through the femoral head assures that all of the debris is out of the joint. Marginally impacted fragments are realigned to the intact femoral head by releasing the traction and using osteotomes and bone graft. Lag screws may help maintain the reduction. A 3.5 mm reconstruction plate is then placed on the medial border of the posterior column, from the sciatic buttress to the ischium, and is fixed with 3.5 mm screws. A spring plate (Figure 2) may be applied in fractures with multiple fragments and small fragments that locate close to the acetabular rim. It is very important to contour the posterior plate precisely to avoid both the anterior gapping of the column and the lack of a buttress of the posterior wall when applying the posterior plate. To avoid avascular necrosis, the posterior wall fragments must not be detached from the capsule. Intraoperative fluoroscopy in multiple views should be used to ensure that all screws are safely placed. An additional lag screw can be placed from the superior aspect of the plate across the transverse fracture line for additional fixation (Figure 9).

#### Tips and tricks

**Figure 10:**
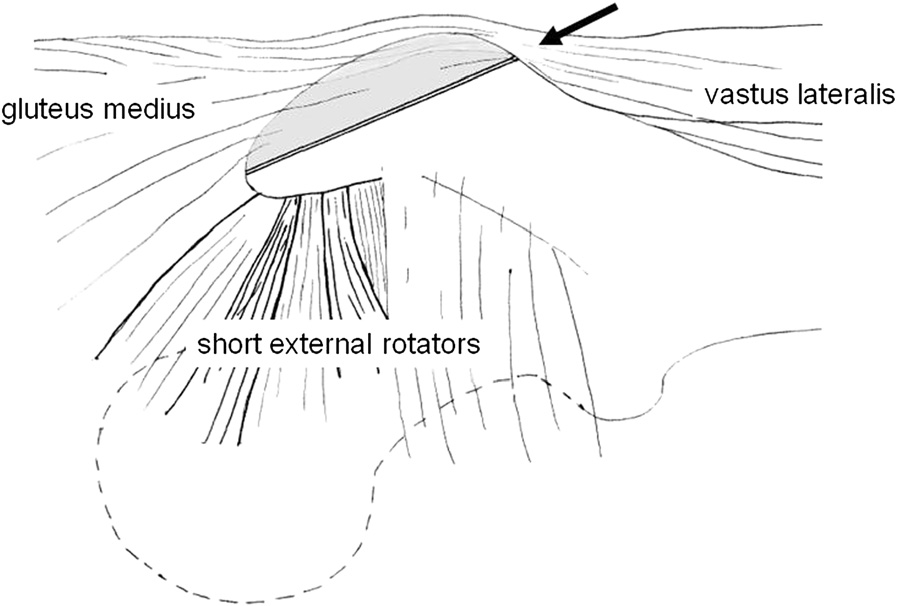
**Trochanter flip approach, as originally described by Reinhold Ganz.** The arrow indicates the osteotomy plane. The gluteus medius and vastus lateralis remain attached to the trochanteric fragment.

If the posterior wall fracture is comminuted and extends through the weight bearing dome, the trochanter flip approach as reported by Reinhold Ganz from Berne, Switzerland, may be useful in addition to the Kocher-Langenbeck approach [16] (Figure 10). This may facilitate the exposure of the superior aspect of the acetabulum, lessen the traction of the superior gluteal vessels, and allow direct vision of the anterior column without a combined or extensile approach. Thus, this may be used in T-shaped fractures as well. Compared with other techniques of the trochanter osteotomy, this approach has several merits such as not detaching vastus lateralis muscle, preserving the blood supply to the femoral head, and less frequency of heterotopic ossification and non-union.

### T-shaped fractures

#### Take home message for safe surgical technique

•*When performing a dual approach, care must be taken to avoid inaccurate fixation of the anterior column from the back and/or posterior column from the front*

•*When indirect reduction of the anterior column is attempted from a posterior approach, the surgeon must be familiar with the placement of instruments into the greater sciatic notch. A ball spike or small bone hook can be gently introduced along the quadrilateral surface to manipulate the anterior column.*

T-shaped fractures are simply transverse fractures with a fracture line separating the anterior column from the posterior column. In these fractures, the posterior capsule is frequently disrupted so there is a need to reduce the two columns separately [17]. This is one of the most difficult fractures to treat surgically, achieving anatomic reduction is difficult, and it tends to have a poorer functional prognosis then the other associated fracture patterns.

#### Surgical approach

Ideally the fracture should be approached, when possible with a single non-extensile incision. The typical T-shaped fracture demonstrates a greater displacement in the posterior column portion of the fracture and the Kocher-Langenbeck approach is common. If the anterior column is more displaced, the ilioinguinal approach may be used. The modified Stoppa approach or the modified ilioinguinal approach can facilitate the visualization of the quadrilateral surface and aid in the visualization and reduction of the posterior column when compared to the standard ilioinguinal approach [10,18]. Ultimately one should strive for a perfect reduction and in some cases based on either experience or fracture pattern it may be necessary to utilize a more extensive surgical approach to achieve the goal. The combination of the anterior and posterior approach may be used, or the extended iliofemoral approach can permit simultaneous exposure and direct control of both columns facilitating the reduction. The extended ilioinguinal exposure is advocated as a primary approach in the following conditions: transtectal fracture line, wide separation of the vertical stem, symphysis displacement, or contralateral rami fractures.

#### Operative procedure

When using the Kocher-Langenbeck approach, the reduction of the posterior column is usually carried out first, ensuring that none of the screws cross into the anterior column fracture segment. The reduction itself is very similar to the pure posterior column fracture. Difficulty is encountered because the lack of a stable anterior column segment. The use of a Schanz screw or two temporary screws with a Faraboeuf clamp may facilitate the reduction and its maintenance. The reduction is checked by the alignment of the greater sciatic notch and at the level of the posterior part of the transverse fracture line dividing the quadrilateral surface by digital palpation. Once reduced, a 3.5 mm reconstruction plate is applied on the lateral border of the greater sciatic notch. The posterior column may be initially fixed with a 3.5 mm lag screw from the intact ilium toward the quadrilateral surface of the fractured posterior column, which allows the removal of the clamps. Care should be taken to assure that no hardware is fixating the anterior column inhibiting its future reduction.

**Figure 11:**
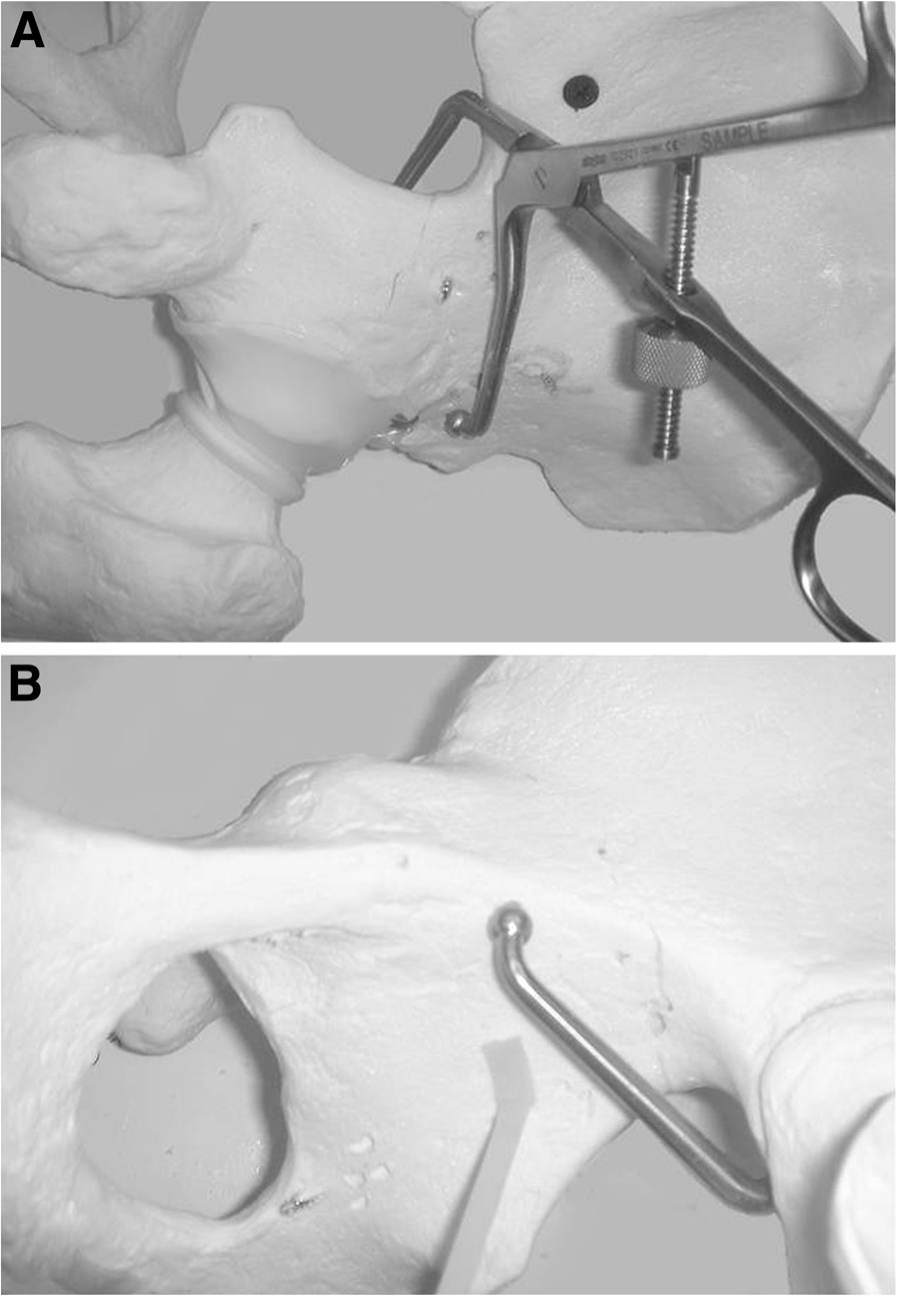
**Pelvic saw bone model demonstrating fracture reduction by the use of an angled reduction clamp.** (**A**) The posterior jaw of the clamp is placed on the above-the-roof area of the intact ilium. (**B**) The anterior jaw is placed on the quadrilateral surface of the fractured anterior column.

**Figure 12:**
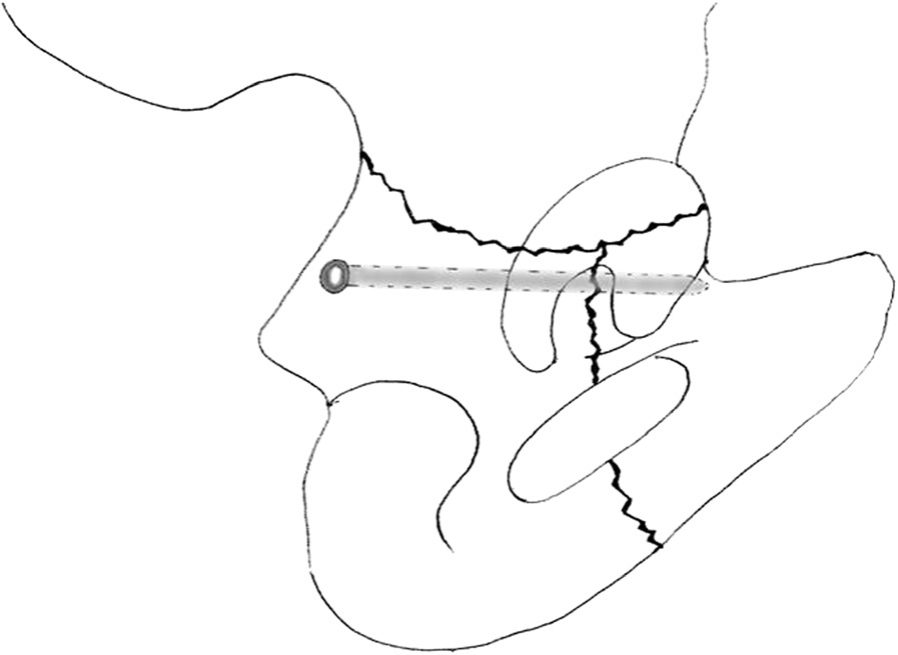
**Schematic drawing of lag screw placement from the posterior to the anterior column.** The screw must be parallel to the quadrilateral surface and checked by intraoperative fluoroscopy to ensure the extraarticular placement.

Indirect reduction of the anterior column is then attempted. The successful reduction of T-shaped fractures through the posterior approach is dependent on this indirect reduction of the anterior column. Thus, the surgeon must be familiar with the placement of instruments into the greater sciatic notch. A ball spike or small bone hook can be gently introduced along the quadrilateral surface to manipulate the anterior column. The technique using an angled reduction clamp through the greater sciatic notch to pull the displaced anterior column distally to fit the intact anterior column and the reconstructed posterior column is also frequently used. One jaw of this clamp should be placed on the quadrilateral surface of the fractured anterior column and the other on the above-the-roof area of the intact ilium, without contacting the posterior column (Figure 11). Traction of the affected lower extremity can help this manipulation. Reduction is confirmed by palpation of the quadrilateral surface, and the anterior column is stabilized using posterior-to-anterior lag screws. This screw starts approximately 3 to 4 cm above the superior edge of the acetabulum and extends directly to the superior pubic ramus as mentioned for associated transverse and posterior wall fractures (Figure 9). However, if possible, the anterior column should be stabilized to the reconstructed posterior column using posterior-to-anterior lag screws. The screw starts from the posterior aspect of the posterior column below the fracture line, directed parallel with the quadrilateral surface, crossing the fracture line, and possibly reaching the pelvic brim (Figure 12). Care must be taken not to injure the anterior neurovascular bundles and not to penetrate the joint. The hip is taken through a range of movements to rule out intra-articular screw penetration. If the reduction of the anterior column is not possible through the posterior approach, a sequential anterior approach can be performed. It is important to assure that no screws are preventing its reduction before changing the approach.

**Figure 13:**
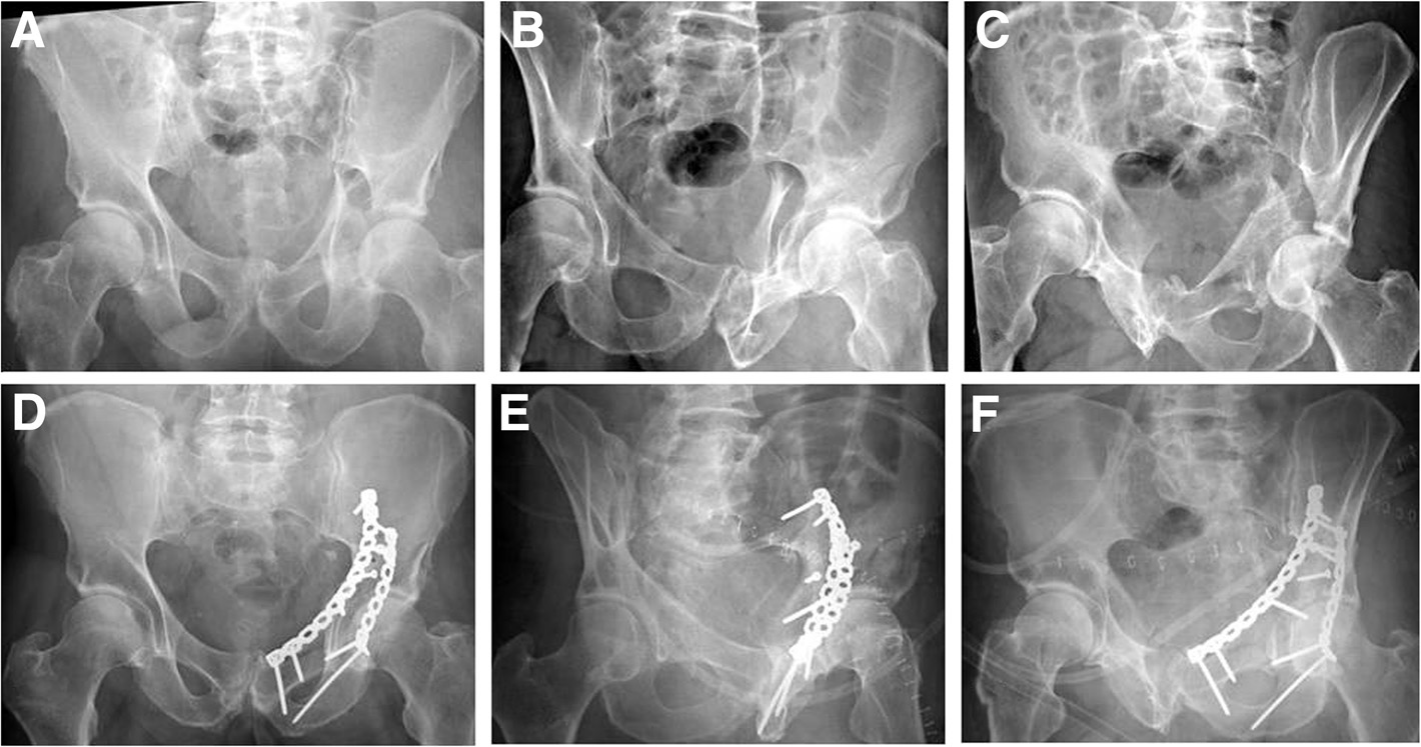
**Example of a T-shaped fracture treated via a combined ilioinguinal and Kocher-Langenbeck approach, using two 3.5 mm reconstruction plates and two 3.5 mm lag screws.** (**A-C**) Preoperative X-rays (a.p., obturator oblique, iliac oblique). (**D-F**) Postoperative X-rays (a.p., obturator oblique, iliac oblique).

When using the anterior approach primarily, the anterior column is reduced first, and indirect reduction of the posterior column is then attempted. The reduction and fixation of the anterior column is the same as in the associated anterior column and posterior hemitransverse fracture. Then the reduction of the posterior column is performed through the quadrilateral surface by using a small bone hook, an asymmetric clamp, or a coaxial pelvic clamp, combined with lateral traction using a Schanz screw in the femoral head. The accuracy of the posterior column reduction may be assessed by inspecting the reduction of the quadrilateral surface to the anterior column. Fixation is carried out using anterior-to-posterior lag screws placed along the pelvic brim, parallel to the quadrilateral surface, and directed toward the ischial spine. These screws may be placed either inside or separate from the pelvic brim plate. If the reduction of the posterior column appears unfeasible, a posterior approach is subsequently performed (Figure 13).

The extended iliofemoral approach can provide complete access to and control of the transverse fracture line. The reduction may be started from either of the columns. They are manipulated and temporarily maintained by means of clamps applied on the outer surface of the innominate bone. The anterior column is usually fixed with a long screw inserted along its axis exactly as described above. The posterior column is fixed with a 3.5 mm reconstruction plate.

#### Tips and tricks

**Figure 14:**
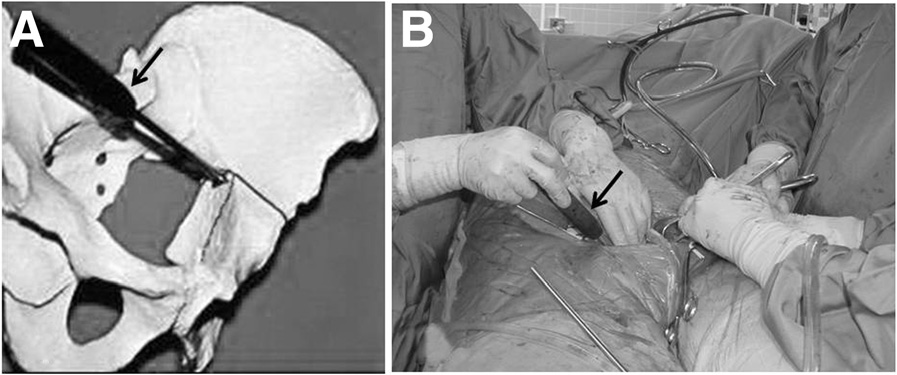
**Reduction of the medially displaced posterior column fragment by pushing the quadrilateral surface through the midline incision with a ball spike pusher (arrow).** (**A**) Saw bone model. (**B**) Intraoperative view.

The modified Stoppa approach developed by Cole et al. [18] or the modified ilioinguinal approach reported by Karunakar et al. [19] that uses the midline incision instead of the third window of the standard ilioinguinal approach, is often useful to treat associated anterior column and posterior hemitransverse, T-shaped, and both column fractures, especially in the case that the quadrilateral surface is not comminuted and the posterior fragment is relative large. This allows access to the pubic symphysis, pubic rami, the whole quadrilateral surface, the inner aspect of the greater sciatic notch, and the sacroiliac joint. Both of the anterior and posterior columns may be directly visualized from the inside of the pelvis. It becomes easier to reduce the medially displaced posterior column fragment by means of pushing the quadrilateral surface through the midline incision with a ball spike (Figure 14). With these approaches, the reduction of the quadrilateral surface may be maintained with lag screws from that surface through the fracture line toward the intact ilium above the greater sciatic notch.

### Both column fractures

#### Take home message for safe surgical technique

•*Intact capsular attachments to both columns usually allow this injury to be approached via and anterior approach with indirect reduction of the posterior column.*

•*Safe fixation of the posterior column from the lateral window of the ilio-inguinal approach can be performed using Judet views to avoid penetration of the hip joint.*

•*In obese patients this antegrade screw fixing the posterior column can be a challenge. Fixation can be achieved by inserting a retrograde screw on a supine patient. Structures at risk in this case are the sciatic nerve which runs just lateral to the entry point (tip of ischial tuberosity) and the para-rectal space medial to it.*

**Figure 15:**
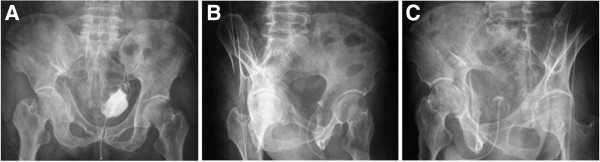
**Example of secondary congruence of an associated both column fracture.** This 56-year-old patient was successfully treated conservatively. (**A**) Anteroposterior view. (**B**) Obturator oblique view. (**C**) Iliac oblique view.

The both column fracture is the most frequent pattern of the associated acetabular fractures. The joint capsule and acetabular labrum, typically, remain firmly attached to both the anterior and posterior column fragments so the fragments can be lined up around the femoral head and the joint surface may appear to be congruent. This phenomenon is known as secondary congruence radiographically, regardless of the medial displacement of femoral head and gaps between articular fragments. Situations of secondary congruence can be managed nonoperatively, but only represent approximately 5% of the fractures [9] (Figure 15). However, the vast majority of these fractures require operative treatment.

#### Surgical approach

This fracture pattern is most frequently treated by the ilioinguinal approach because it often allows the reduction from within the pelvis by hinging fragments on their remaining capsular attachment. Reduction of the anterior column to create a stable surface to reconstruct the remaining articular surface is the key in the reconstruction of this fracture pattern [18]. This is generally best performed by an exposure that allows extensive exposure to this aspect of the ilium. If the fracture involves a complex fracture of the quadrilateral surface that is separated from the posterior column, a displaced fracture line crossing the sacroiliac joint, or a wide separation between the anterior and posterior column fracture, either the combination of the anterior and posterior approach or the extended iliofemoral approach is appropriate.

#### Operative procedure

The key to reconstruction is anatomic reconstruction of the anterior column. Thus, the first step in the procedure is fixation of the large anterior fragment to the intact ilium, and it is necessary to attempt to restore the normal concavity of the iliac fossa which is much greater than what appears under direct visualization. The anterior column is usually rotated externally and shortened, and the reduction is carried out using a ball spike pusher placed above the pelvic brim on the intact iliac fossa. An asymmetric reduction forceps placed across the iliac brim and a Farabeuf clamp placed at the level of the iliac crest may also be advantageous in obtaining and maintaining the reduction of the anterior column. The femoral head typically follows the anterior column fragment and should become reduced following the reduction of the anterior column. If there is a triangular fragment along the iliac crest or a posterior fragment of the pelvic brim, it should be reduced and fixed accurately, perhaps first to the posterior part of the iliac wing. These provide an anatomic template for the subsequent reduction of the posterior column. Digital palpation of the outer table of the ilium and the fracture line should be undertaken to insure that there is no malreduction of the anterior column. A small malreduction in the ilium can result in a significant step off at the articualr surface. This is made possible by elevating the soft tissue along each side of the fracture line of the iliac crest. The fixation of the iliac crest is achieved by inserting 3.5 mm or 4.5 mm lag screws. Fixation is largely obtained as previously described for anterior column posterior hemitransverse fractures.

**Figure 16:**
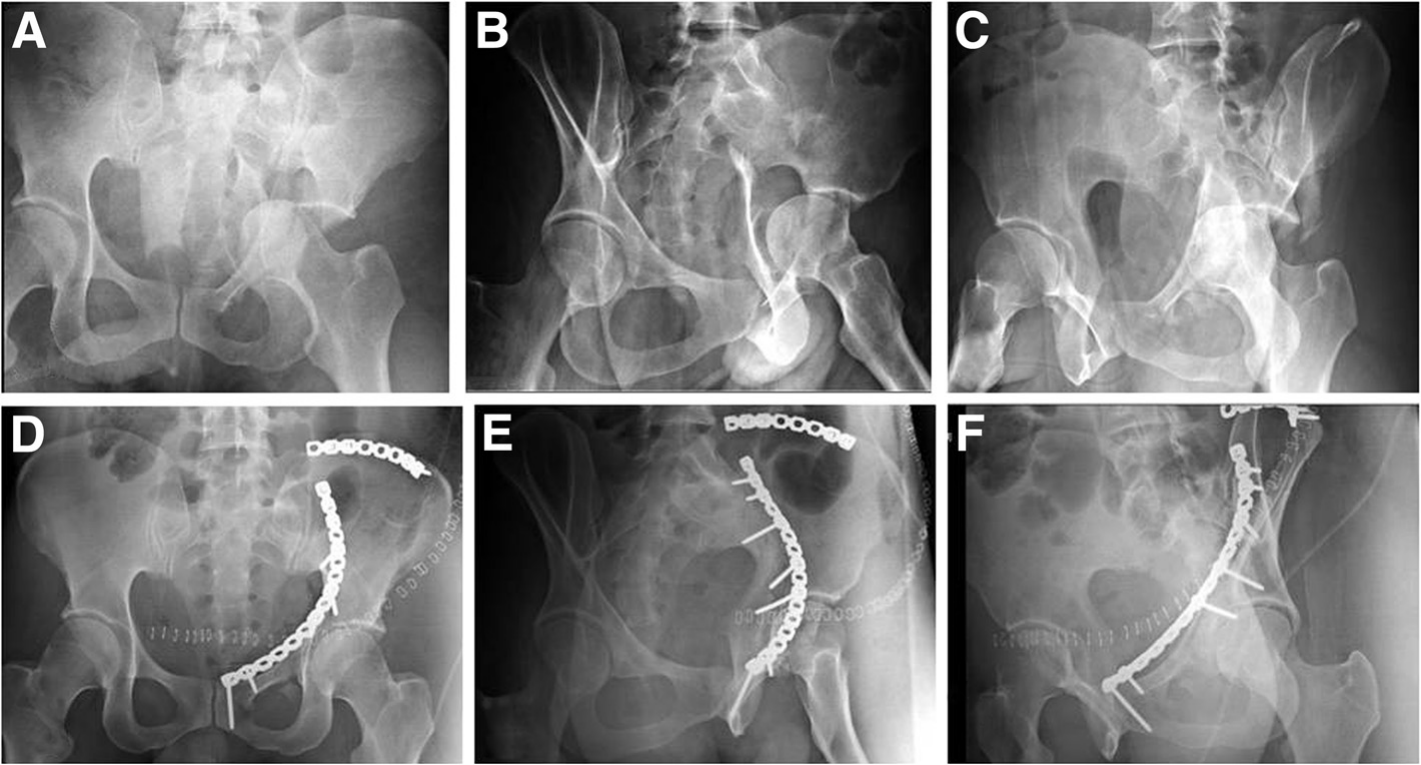
**Example of an associated both column fracture treated through a modified ilioinguinal approach with two 3.5 mm reconstruction plates.** (**A-C**) Preoperative X-rays (a.p., obturator oblique, iliac oblique). (**D-F**) Postoperative X-rays (a.p., obturator oblique, iliac oblique).

Reduction of the posterior column can be facilitated by placement of a Schanz screw in the femoral neck for anterior and lateral traction either manually or with the use of a traction table. Pelvic reduction clamps, with one jaw on the outer surface of the anterior inferior iliac spine and the other jaw through the first or second window on the quadrilateral surface of the posterior column, help achieve reduction. Reduction may also be achieved by means of a small bone hook or a coaxial pelvic reduction clamp. The posterior column is stabilized using anterior-to-posterior lag screws, some of which may pass through plate holes placed on the pelvic brim. These screws start at the pelvic brim 3 to 5 cm anterior to the sacroiliac joint and are directed from proximal to distal into the posterior column paralleling the quadrilateral surface, aiming for the ischial spine. Joint penetration is likely to occur with these screws. The reduction is checked radiographically and with digital palpation of the accessible fracture surfaces (Figure 16). If the reduction of the posterior column is not possible through the anterior approach, the sequential Kocher-Langenbeck approach can be performed. In this setting, anterior implants must be carefully positioned not to impede subsequent reduction from the secondary posterior approach.

**Figure 17:**
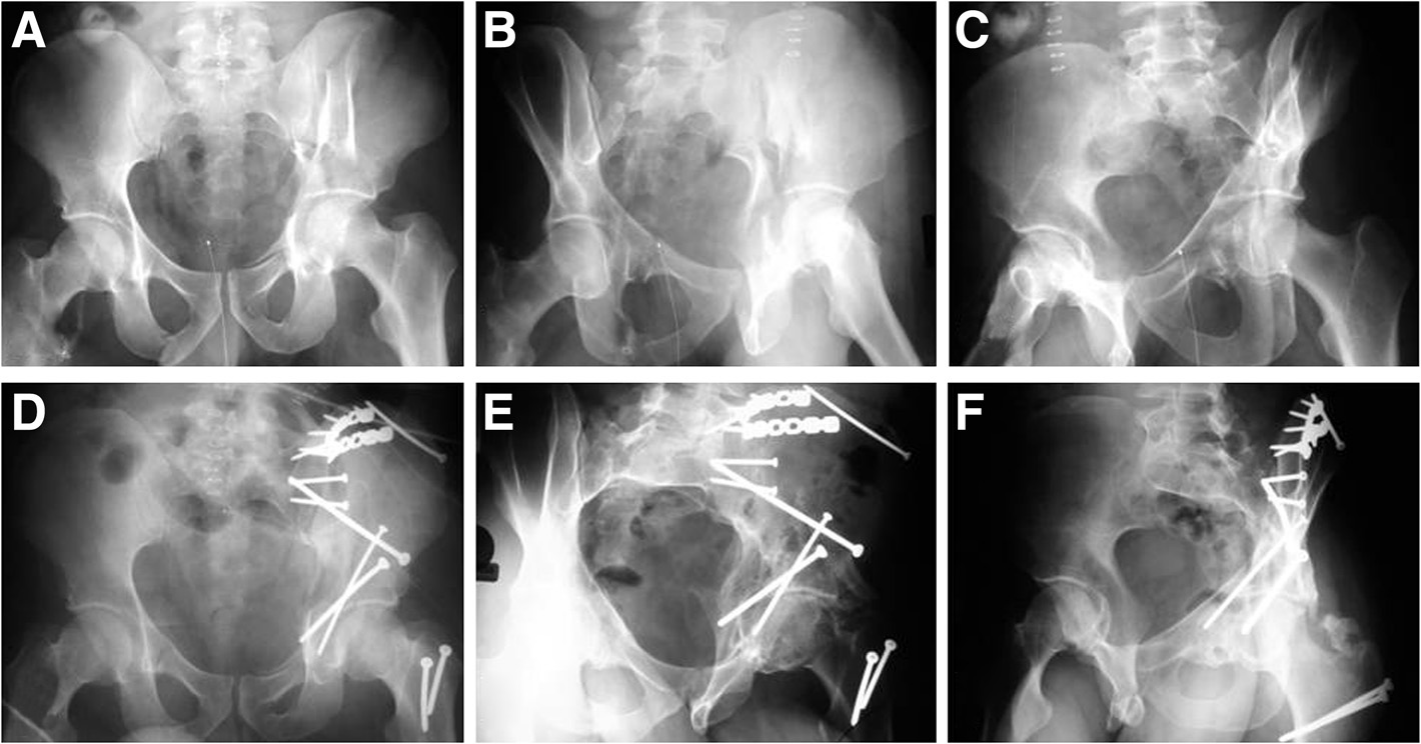
**Example of an associated both column fracture treated through an extended iliofemoral approach, with a fracture line extending into the sacroiliac joint.** (**A-C**) Preoperative X-rays (a.p., obturator oblique, iliac oblique). (**D-F**) Postoperative X-rays (a.p., obturator oblique, iliac oblique).

When the extended iliofemoral approach is selected (Figure 17), the whole posterior column, the whole iliac wing, and the anterior column up to the iliopectineal eminence can be visually inspected. The internal aspect of the iliac fossa can be digitally inspected for fracture reduction. Care must be taken to avoid stripping the internal iliac fossa so as to not devitalize the anterior column segment. The order of fixation remains the same as with an anterior based approach.

#### Tips and tricks

**Figure 18:**
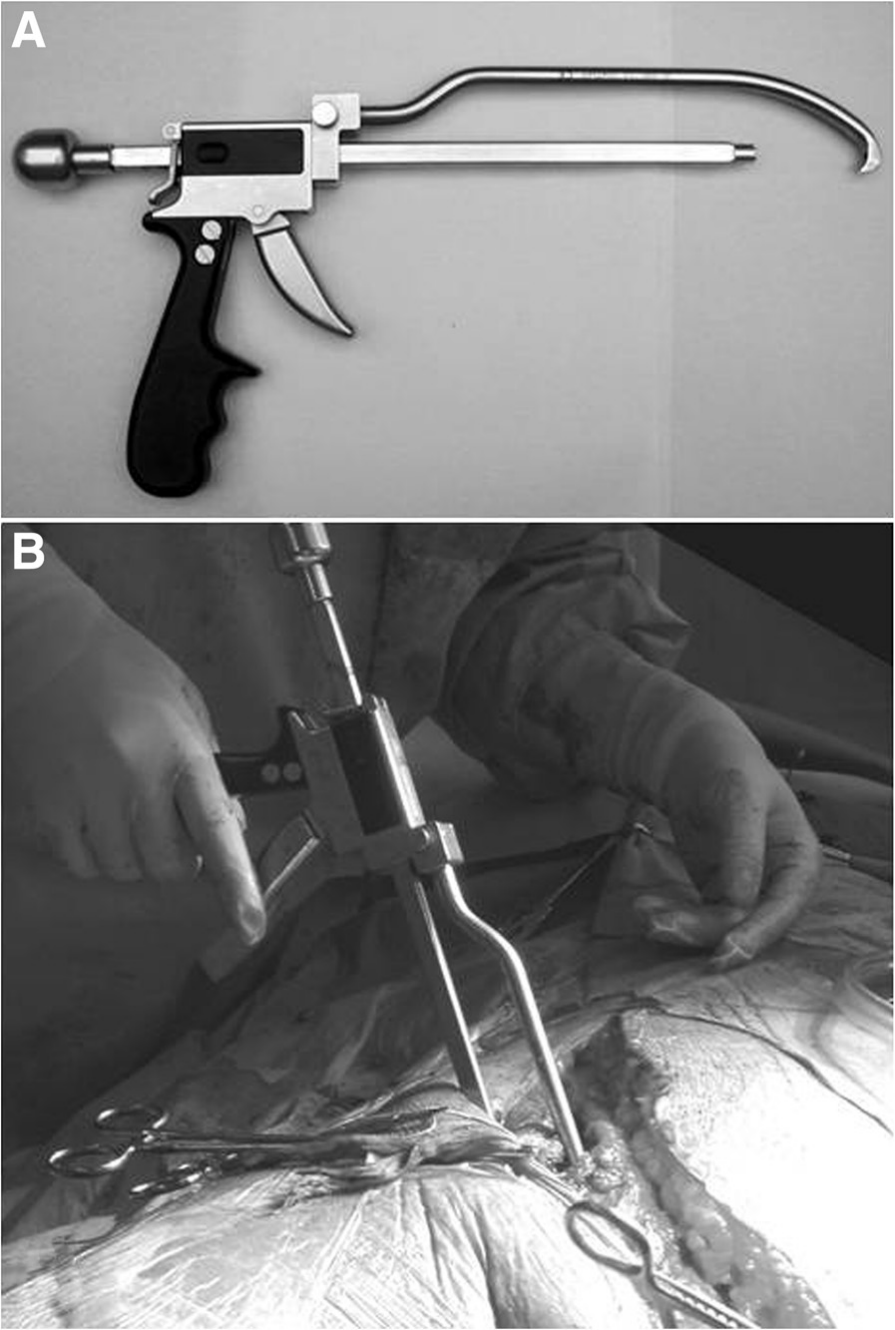
**The AO coaxial pelvic clamp.** (**A**) This clamp can pull the posterior column up to anterior column by pulling the trigger like a “gun”. (**B**) Application through a small window of ilioinguinal approach.

The AO coaxial pelvic clamp is designed to control fragments through the small window via the axial sliding mechanism of its forceps. It can be useful to reduce the posterior column, especially those posteriorly displaced in the both column fractures and T-shaped fractures, through the anterior approach. The jaw located proximal to this clamp is placed on the pelvic brim with its tip anchored on the posterior edge of the quadrilateral surface or ischial spine, and can pull the posterior column up to anterior column by pulling the trigger like a gun (Figure 18).

## Conclusion

In this paper we present specific tips and tricks for the safe surgical management of associated acetabular fractures. The success to satisfactory outcome is to aim for Letournel’s so-called *“reduction parfaite”*. In order to achieve this, a number of steps must be followed:

Adequate preoperative imaging and preoperative planning

Choice of surgical approach and, if necessary, a staged management

Good quality intraoperative fluoroscopy views to confirm perfect reduction and extra-articular placement of hardware.

A perfect knowledge of pelvic and acetabular anatomy is essential to prevent potentially lethal complications and a allow safe reduction and fixation.

## Competing interests

The authors declare no conflict of interest related to this manuscript. All authors confirm that they have no financial relationship to companies involved in the marketing of the products described in this article.

## Authors’ contributions

WRS and SJM designed the concept of this article. TS drafted the first version of the manuscript. CM reviewed and edited the final version of the manuscript. All authors read and approved the final version of this manuscript.

## References

[B1] Judet R Judet J Letournel E **Fractures of the acetabulum; classification and surgical approaches for open reduction.** Preliminary report J Bone Joint Surg Am 1964 46 1615 1646 14239854

[B2] Helfet DL Schmeling GJ Management of complex acetabular fractures through single nonextensile exposures Clin Orthop Relat Res 1994 305 58 68 8050248

[B3] Mears DC Velyvis JH Chang CP Displaced acetabular fractures managed operatively: indicators of outcome Clin Orthop Relat Res 2003 407 173 186 12567145 10.1097/00003086-200302000-00026

[B4] Matta JM Merritt PO Displaced acetabular fractures Clin Orthop Relat Res 1988 230 83 97 3365902

[B5] Olson SA Matta JM The computerized tomography subchondral arc: a new method of assessing acetabular articular continuity after fracture: a preliminary report J Orthop Trauma 1993 7 402 413 10.1097/00005131-199310000-00002 8229376

[B6] Mears DC Velyvis JH Acute total hip arthroplasty for selected displaced acetabular fractures: two to twelve-year results J Bone Joint Surg Am 2002 84 1 9 10.1302/0301-620X.84B1.12792 11792772

[B7] Matta JM Fractures of the acetabulum: accuracy of reduction and clinical results in patients managed operatively within three weeks after the injury J Bone Joint Surg Am 1996 78 1632 1645 8934477

[B8] Goulet JA Rouleau JP Mason DJ Goldstein SA **Comminuted fractures of the posterior wall of the acetabulum.** A biomechanical evaluation of fixation methods J Bone Joint Surg Am 1994 76 1457 1463 7929492 10.2106/00004623-199410000-00004

[B9] Matta JM Operative indications and choice of surgical approach for fractures of the acetabulum Tech Orthop 1986 1 13 10.1097/00013611-198604000-00006

[B10] Ponsen KJ Joosse P Schigt A Goslings JC Luitse JS Internal fracture fixation using the Stoppa approach in pelvic ring and acetabular fractures: technical aspects and operative results J Trauma 2006 61 662 667 10.1097/01.ta.0000219693.95873.24 16967004

[B11] Keel MJ Ecker TM Cullmann JL Bergmann M Bonel HM Büchler L Siebenrock KA Bastian JD The Pararectus approach for anterior intrapelvic management of acetabular fractures: anatomical study and clinical evaluation J Bone Joint Surg Br 2012 94 405 11 10.1302/0301-620X.94B3.27801 22371551

[B12] Ebraheim NA Xu R Biyani A Benedetti JA Anatomic basis of lag screw placement in the anterior column of the acetabulum Clin Orthop Relat Res 1997 339 200 205 9186221 10.1097/00003086-199706000-00028

[B13] Simonian PT Routt ML Jr Harrington RM Tencer AF The unstable iliac fracture: a biomechanical evaluation of internal fixation Injury 1997 28 469 475 10.1016/S0020-1383(97)00112-5 9509089

[B14] Reddix RN Jr Webb LX Computer-assisted preoperative planning in the surgical treatment of acetabular fractures J Surg Orthop Adv 2007 16 138 143 17963657

[B15] Hirvensalo E Lindahl J Kiljunen V Modified and new approaches for pelvic and acetabular surgery Injury 2007 38 431 441 10.1016/j.injury.2007.01.020 17445529

[B16] Ganz R Gill TJ Gautier E Ganz K Krugel N Berlemann U Surgical dislocation of the adult hip a technique with full access to the femoral head and acetabulum without the risk of avascular necrosis J Bone Joint Surg Br 2001 83 1119 1124 10.1302/0301-620X.83B8.11964 11764423

[B17] Vrahas MS Widding KK Thomas KA The effects of simulated transverse, anterior column, and posterior column fractures of the acetabulum on the stability of the hip joint J Bone Joint Surg Am 1999 81 966 974 10428128 10.2106/00004623-199907000-00009

[B18] Cole JD Bolhofner BR **Acetabular fracture fixation via a modified Stoppa limited intrapelvic approach.** Description of operative technique and preliminary treatment results Clin Orthop Relat Res 1994 305 112 123 8050220

[B19] Karunakar MA Le TT Bosse MJ The modified ilioinguinal approach J Orthop Trauma 2004 18 379 383 10.1097/00005131-200407000-00009 15213504

